# Supplementary material for: Metabolic stress and muscle mechanics: Acute response of isolated soleus and EDL muscles to prolonged fasting in mice with distinct muscle phenotypes
Source: Biol Open. 2025 Oct 21;14(10):bio062245. doi: 10.1242/bio.062245 (PMC12584392; doi:10.1242/bio.062245)
Supplement: Supplementary information [file biolopen-14-062245-s1.pdf]

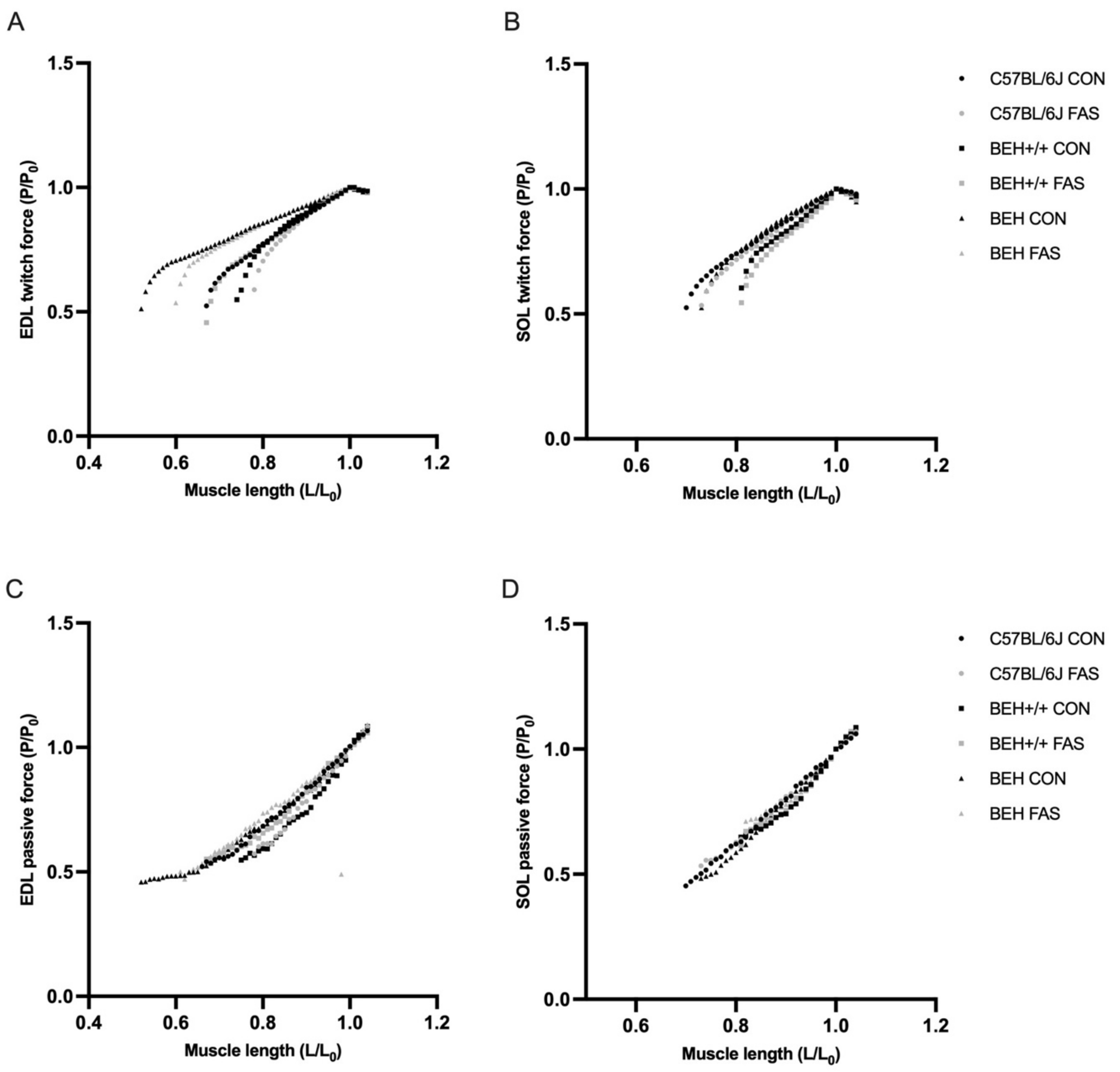

Fig. S1. Representative active and passive force–length traces from extensor digitorum longus (EDL) and soleus (SOL) muscles. (A–B) Active force normalized to peak active force (P_0). (C–D) Passive force normalized to peak passive force. Data are shown from a single muscle per group as indicative examples to illustrate the protocol. The curves were obtained to determine optimal length (L_0) for subsequent testing.

Biology Open • Supplementary information

Table S1. Isometric (ISO) and eccentric (ECC) contractile properties of extensor digitorum longus (EDL) and soleus (SOL) muscles across the contraction cycles in control (CON) and fasting (FAS) mice of different strains.

	C57BL/6J																	
	1st contraction				10th contraction				50th contraction				100th contraction					
	EDL		SOL		EDL		SOL		EDL		SOL		EDL		SOL		*Main effect p*	*Interaction p*
	CON	*FAS*	*CON*	*FAS*	*CON*	*FAS*	*CON*	*FAS*	*CON*	*FAS*	*CON*	*FAS*	*CON*	*FAS*	*CON*	*FAS*		
PF_{ISO} (mN)	188 ± 28.3	133 ± 32.9	165 ± 14.5	107 ± 20.4	123 ± 16.2	82.3 ± 25.1	120 ± 17.9	78.4 ± 14.2	76.1 ± 11.8	43.6 ± 11.0	95.5 ± 10.4	59.2 ± 10.2	70.9 ± 11.8	40.6 ± 10.9	94.9 ± 13.7	55.2 ± 14.1		
P_0ISO (mN/mm^2)	110 ± 23.2	87.6 ± 19.6	167 ± 20.0	122 ± 28.1	69.2 ± 10.8	53.2 ± 13.3	120 ± 17.3	88.1 ± 20.9	42.7 ± 7.88	28.3 ± 5.29	95.9 ± 14.0	66.5 ± 15.3	39.8 ± 8.24	26.3 ± 5.29	95.0 ± 14.0	63.0 ± 10.3		
RFD (mN × ms^{-1})	1660 ± 465	1066 ± 276	474 ± 38.4	264 ± 40.8	1545 ± 600	940 ± 478	409 ± 32.7	214 ± 51.4	1028 ± 649	713 ± 568	342 ± 22.7	191 ± 28.6	774 ± 437	520 ± 333	323 ± 22.6	183 ± 27.4	ttt, cc, ss, mm	SC, CM, SMT
sRFD (mN/mm2 × ms^{-1})	976 ± 321	705 ± 180	479 ± 56.4	301 ± 61.6	871 ± 361	602 ± 272	411 ± 46.1	247 ± 72.5	582 ± 378	451 ± 334	344 ± 43.4	218 ± 50.7	438 ± 307	333 ± 154	324 ± 47.7	210 ± 26.5		
RFD/PF (%)	8.70 ± 1.30	8.03 ± 0.78	2.88 ± 0.17	2.49 ± 0.23	12.2 ± 3.27	10.9 ± 2.48	3.44 ± 0.34	3.06 ± 3.06	12.8 ± 6.14	14.9 ± 8.48	3.61 ± 0.27	3.61 ± 0.51	10.7 ± 6.82	12.2 ± 3.45	3.44 ± 0.18	3.61 ± 0.10		
PF_{ECC} (mN)	383 ± 55.2	277 ± 50.3	384 ± 20.2	271 ± 46.7	327 ± 50.9	221 ± 43.0	327 ± 18.9	263 ± 79.9	212 ± 31.5	157 ± 17.4	282 ± 17.9	231 ± 25.7	209 ± 37.4	149 ± 17.2	268 ± 17.6	225 ± 42.5		
SIFE (mN)	196 ± 28.0	144 ± 18.2	217 ± 17.3	150 ± 35.0	203 ± 34.7	139 ± 18.9	207 ± 11.2	185 ± 17.0	136 ± 20.6	113 ± 9.27	186 ± 9.7	172 ± 17.7	138 ± 28.3	109 ± 9.28	173 ± 19.0	165 ± 29.4	ttt, ccc, s, m	ns
RFE (mN)	-182 ± 31.1	-123 ± 34.2	-144 ± 14.9	-93.5 ± 19.6	-119 ± 16.6	-70.0 ± 23.6	-106 ± 13.4	-61.6 ± 13.1	-72.5 ± 13.5	-37.2 ± 11.7	-86 ± 8.1	-47.1 ± 9.6	-67.4 ± 12.6	-35.5 ± 14.0	-85.1 ± 9.7	-45.6 ± 11.3		

	BEH+/+															
	1st contraction				10th contraction				50th contraction				100th contraction			
	EDL		SOL		EDL		SOL		EDL		SOL		EDL		SOL	
	CON	*FAS*	*CON*	*FAS*	*CON*	*FAS*	*CON*	*FAS*	*CON*	*FAS*	*CON*	*FAS*	*CON*	*FAS*	*CON*	*FAS*
PF_{ISO} (mN)	228 ± 20.0	200 ± 13.7	210 ± 19.2	151 ± 29.6	143 ± 9.45	107 ± 47.6	165 ± 17.7	101 ± 22.5	93.0 ± 4.84	64.0 ± 29.4	125 ± 14.7	85.1 ± 15.3	70.6 ± 17.7	54.0 ± 25.9	105 ± 19.5	79.0 ± 16.3
P_0ISO (mN/mm^2)	115 ± 13.9	98.4 ± 8.26	182 ± 16.3	181 ± 45.6	69.9 ± 4.38	51.7 ± 22.9	144 ± 17.8	90 ± 23.0	40.8 ± 6.84	30.9 ± 14.3	108 ± 16.1	75.3 ± 16.3	32.2 ± 11.2	25.9 ± 12.1	91.7 ± 11.0	68.5 ± 6.5
RFD (mN × ms^{-1})	1892 ± 232	1525 ± 107	526 ± 49.5	526 ± 49.5	1766 ± 193	1352 ± 653	504 ± 43.4	343 ± 48.4	1141 ± 88	873 ± 93	395 ± 37.5	302 ± 31.6	872 ± 404	718 ± 347	366 ± 42.7	287 ± 31.0
sRFD (mN/mm2 × ms^{-1})	955 ± 177	750 ± 51.9	455 ± 45.6	455 ± 45.6	861 ± 86.5	653 ± 279	440 ± 42.0	308 ± 52.8	500 ± 93	422 ± 197	345 ± 39.7	271 ± 38.9	404 ± 171	347 ± 157	320 ± 43.2	257 ± 25.5
RFD/PF (%)	8.31 ± 0.91	7.65 ± 0.56	2.50 ± 0.10	2.43 ± 0.16	12.3 ± 0.654	13.2 ± 1.51	3.06 ± 0.12	3.03 ± 0.23	12.3 ± 0.66	14.1 ± 1.73	3.18 ± 0.12	3.17 ± 0.22	11.8 ± 2.68	14.3 ± 2.60	3.50 ± 0.16	3.29 ± 0.23
PF_{ECC} (mN)	453 ± 33.5	394 ± 28.0	496 ± 38.4	360 ± 52.5	375 ± 34.8	292 ± 108	445 ± 40.1	265 ± 60.7	261 ± 14.1	193 ± 63.6	376 ± 32.0	247 ± 42.4	202 ± 38.5	165 ± 50.1	336 ± 56.3	228 ± 47.6
SIFE (mN)	225 ± 19.2	119 ± 49.0	314 ± 33.9	283 ± 32.5	232 ± 27.4	185 ± 61.3	280 ± 24.3	165 ± 39.3	168 ± 10.6	129 ± 34.7	251 ± 19.6	155 ± 29.3	131 ± 21.1	111 ± 26.2	231 ± 47.9	150 ± 32.9
RFE (mN)	-224 ± 21.7	-194 ± 16.6	-182 ± 18.8	-135 ± 32.5	-141 ± 8.60	-104 ± 45.0	-146 ± 14.6	-101 ± 24.3	-88.4 ± 6.04	-49.3 ± 38.8	-112 ± 13.9	-87.7 ± 13.7	-68.3 ± 16.9	-51.7 ± 22.5	-95.9 ± 12.4	-80.8 ± 13.6

	BEH															
	1st contraction				10th contraction				50th contraction				100th contraction			
	EDL		SOL		EDL		SOL		EDL		SOL		EDL		SOL	
	CON	*FAS*	*CON*	*FAS*	*CON*	*FAS*	*CON*	*FAS*	*CON*	*FAS*	*CON*	*FAS*	*CON*	*FAS*	*CON*	*FAS*
PF_{ISO} (mN)	316 ± 27.2	291 ± 39.1	267 ± 43.4	175 ± 13.0	178 ± 28.8	166 ± 33.7	172 ± 27.6	106 ± 14.5	94.3 ± 23.1	85.4 ± 16.8	122 ± 16.4	94 ± 16.9	66.5 ± 12.4	57.0 ± 22.6	110 ± 16.1	80.0 ± 16.3
P_0ISO (mN/mm^2)	85.3 ± 9.83	81.2 ± 9.71	144 ± 23.2	98 ± 12.0	58.1 ± 12.4	41.9 ± 6.47	90.2 ± 9.9	59.6 ± 8.8	23.1 ± 5.48	19.0 ± 2.81	64.2 ± 5.2	52.1 ± 10.1	14.7 ± 2.41	14.0 ± 5.41	57.7 ± 5.1	49.5 ± 6.5
RFD (mN × ms^{-1})	3300 ± 196	2885 ± 331	722 ± 109.0	473 ± 30.8	2234 ± 725	2037 ± 371	607 ± 110.6	400 ± 177.0	1117 ± 274	1068 ± 200	416 ± 59.9	348 ± 156.0	782 ± 172	581 ± 140	405 ± 67.6	302 ± 68.6
sRFD (mN/mm2 × ms^{-1})	848 ± 81.5	832 ± 84.8	391 ± 58.1	264 ± 30.8	725 ± 155	513 ± 79.3	320 ± 72.5	222 ± 30.1	274 ± 93	239 ± 41	219 ± 50.7	192 ± 21.5	172 ± 26.6	331 ± 78.8	213 ± 56.0	167 ± 38.4
RFD/PF (%)	10.5 ± 0.42	9.77 ± 0.42	2.71 ± 0.13	2.70 ± 0.23	12.5 ± 0.26	12.3 ± 1.33	3.54 ± 0.34	3.19 ± 0.23	11.9 ± 0.17	12.5 ± 0.35	3.41 ± 0.14	3.19 ± 0.39	11.7 ± 0.301	9.63 ± 2.69	3.69 ± 0.20	3.18 ± 0.17
PF_{ECC} (mN)	597 ± 25.4	543 ± 62.9	648 ± 91.8	451 ± 25.4	428 ± 82.2	407 ± 46.7	523 ± 79.9	285 ± 80.3	268 ± 15.8	263 ± 30.5	400 ± 45.1	249 ± 45.7	216 ± 16.6	174 ± 56.5	366 ± 54.1	235 ± 47.6
SIFE (mN)	281 ± 16.1	246 ± 25.6	381 ± 49.8	276 ± 44.6	249 ± 52.3	240 ± 30.2	351 ± 54.0	180 ± 18.3	173 ± 9.92	178 ± 14.4	278 ± 29.5	154 ± 30.5	150 ± 5.65	117 ± 34.3	256 ± 39.6	156 ± 27.9
RFE (mN)	-313 ± 26.3	-294 ± 39.2	-231 ± 38.8	-159 ± 13.6	-169 ± 31.4	-164 ± 27.9	-152 ± 20.9	-116 ± 14.0	-91.9 ± 8.46	-83.1 ± 15.9	-113 ± 13.8	-105 ± 18.1	-64.4 ± 11.5	-55.8 ± 22.4	-103 ± 20.5	-89.0 ± 14.4

EDL: extensor digitorum longus; SOL: soleus; CON: control condition; FAS: fasting condition; ISO: isometric; ECC: eccentric; PF, peak force; P0: specific force; RFD; rate of force development; SIFE, stretch-induced force enhancement; RFE: residual force enhancement . Notes: ttt, time effect $p<0.001$; s, strain effect $p<0.05$; ss, strain effect $p<0.01$; cc, condition effect $p<0.01$; ccc, condition effect $p<0.001$; m, muscle type effect $p<0.05$; mm, muscle type effect $p<0.01$; SC strain x condition interaction $p<0.05$; CM, condition x muscle type interaction $p<0.05$; SMT, strain × muscle type × time interaction $p<0.05$.

Table 2. Eccentric (ECC) biomechanical properties of extensor digitorum longus (EDL) and soleus (SOL) muscles across the 1st, 10th, 50th, and 100th contraction cycles in control (CON) and fasting (FAS) mice of different strains.

	1st contraction				10th contraction				50th contraction				100th contraction					
**C57BL/6J**	EDL		SOL		EDL		SOL		EDL		SOL		EDL		SOL		***Main effect p***	***Interaction p***
	CON	*FAS*	*CON*	*FAS*	*CON*	*FAS*	*CON*	*FAS*	*CON*	*FAS*	*CON*	*FAS*	*CON*	*FAS*	*CON*	*FAS*		
ST_{ECC} (mN/mm)	100.3 ± 9.2	89.3 ± 7.13	79.6 ± 5.80	61.7 ± 6.50	114.2 ± 14.8	88.5 ± 13.3	79.2 ± 5.10	62.3 ± 8.20	77.6 ± 9.68	69.4 ± 16.19	70.3 ± 3.92	55.9 ± 8.68	72.3 ± 13.8	67.8 ± 16.8	66.8 ± 3.04	53.5 ± 7.22	ttt, ccc, ss	SC
TM_{ECC} (MPa)	8.36 ± 1.54	7.91 ± 0.44	10.33 ± 1.20	8.83 ± 2.00	9.27 ± 1.5	8.72 ± 1.0	10.3 ± 1.50	9.62 ± 1.40	6.74 ± 1.14	6.19 ± 1.40	8.33 ± 1.10	7.58 ± 1.23	5.75 ± 1.46	6.61 ± 1.45	8.87 ± 1.07	7.37 ± 1.16		
**BEH+/+**	1st contraction				10th contraction				50th contraction				100th contraction					
	EDL		SOL		EDL		SOL		EDL		SOL		EDL		SOL			
	CON	*FAS*	*CON*	*FAS*	*CON*	*FAS*	*CON*	*FAS*	*CON*	*FAS*	*CON*	*FAS*	*CON*	*FAS*	*CON*	*FAS*		
ST_{ECC} (mN/mm)	108.6 ± 6.1	95.4 ± 5.8	97.6 ± 8.5	72.0 ± 7.1	119.7 ± 15.5	97.3 ± 31.8	95.2 ± 8.8	70.75 ± 5.53	87.5 ± 5.66	68.9 ± 18.6	88.5 ± 5.44	66.2 ± 5.35	74.6 ± 10.7	61.5 ± 13.0	82.3 ± 4.33	65.5 ± 6.13		
TM_{ECC} (MPa)	8.57 ± 1.2	7.89 ± 1.5	11.67 ± 1.0	9.45 ± 0.7	8.10 ± 1.8	7.42 ± 2.5	11.25 ± 1.12	9.18 ± 0.75	6.25 ± 1.07	5.55 ± 2.45	10.77 ± 1.12	9.01 ± 0.73	5.06 ± 1.32	4.25 ± 1.86	10.55 ± 1.23	8.95 ± 0.87		
**BEH**	1st contraction				10th contraction				50th contraction				100th contraction					
	EDL		SOL		EDL		SOL		EDL		SOL		EDL		SOL			
	CON	*FAS*	*CON*	*FAS*	*CON*	*FAS*	*CON*	*FAS*	*CON*	*FAS*	*CON*	*FAS*	*CON*	*FAS*	*CON*	*FAS*		
ST_{ECC} (mN/mm)	119.5 ± 7.11	104.2 ± 5.51	119 ± 15.8	87.2 ± 3.60	125.2 ± 13.2	102.1 ± 17.9	116.8 ± 21.0	82.8 ± 5.00	83.0 ± 4.55	65.1 ± 3.41	92.0 ± 10.4	63.0 ± 5.28	73.0 ± 1.59	55.7 ± 16.7	84.0 ± 7.49	56.0 ± 6.30		
TM_{ECC} (MPa)	5.30 ± 0.45	4.95 ± 0.54	9.73 ± 2.00	6.89 ± 0.50	5.56 ± 0.30	4.81 ± 0.80	9.62 ± 1.50	6.79 ± 0.60	4.52 ± 0.09	3.56 ± 0.16	7.92 ± 0.56	5.94 ± 0.69	4.04 ± 0.20	3.01 ± 0.56	7.15 ± 0.42	5.06 ± 0.79		

EDL: extensor digitorum longus; SOL: soleus; CON: control condition; FAS: fasting condition; ECC: eccentric; ST: stiffness; TM: tangent modulus. Notes: ttt, time effect $p<0.001$; s, strain effect $p<0.05$; ss, strain effect $p<0.01$; c, condition effect $p<0.05$; ccc, condition effect $p<0.001$; SC strain x condition interaction $p<0.05$.

Table S3. Final isometric force production variables of extensor digitorum longus (EDL) and soleus (SOL) muscles in control (CON) and fasting (FAS) mice of different strains.

	C57BL/6J				Main effect p	Interaction p
	EDL		SOL			
	CON	*FAS*	*CON*	*FAS*		
PF_{ISO} (mN)	83.7 ± 13.3	74.3 ± 10.4	115 ± 12.6	78.3 ± 8.97		
P_0ISO (mN/mm^2)	46.2 ± 9.1	40.1 ± 13.3	115 ± 16.6	90.3 ± 17.1		
RFD (mN × ms^{-1})	1064 ± 202	906 ± 209	339 ± 33.7	211 ± 33.6	cc, ss, mm	SC, CM, SMT
sRFD (mN/mm2 × ms^{-1})	598 ± 134	416 ± 138	340 ± 45.7	244 ± 58.8		
RFD/PF (%)	13.5 ± 0.8	10.1 ± 7.7	2.96 ± 0.33	2.69 ± 0.24		
FI (%)	48.3 ± 4.3	39.7 ± 9.9	70.7 ± 5.01	76.7 ± 9.19		
	BEH+/+					
	EDL		SOL			
	CON	*FAS*	*CON*	*FAS*		
PF_{ISO} (mN)	76.8 ± 8.6	66.1 ± 10.9	131 ± 19.7	111 ± 9.94		
P_0ISO (mN/mm^2)	44.1 ± 9.9	34.3 ± 16.4	69.1 ± 6.8	61.4 ± 6.5		
RFD (mN × ms^{-1})	597 ± 57.8	478 ± 7.7	396 ± 46.4	319 ± 25.2		
sRFD (mN/mm2 × ms^{-1})	385 ± 131	269 ± 148	209 ± 14.2	176 ± 14.2		
RFD/PF (%)	10.6 ± 1.0	8.9 ± 7.7	3.03 ± 0.16	2.88 ± 0.23		
FI (%)	39.8 ± 16.1	28.9 ± 14.7	53.1 ± 11.5	65.6 ± 3.75		
	BEH					
	EDL		SOL			
	CON	*FAS*	*CON*	*FAS*		
PF_{ISO} (mN)	79.3 ± 14.8	70.1 ± 7.7	137 ± 16.3	112 ± 20.2		
P_0ISO (mN/mm^2)	20.4 ± 2.9	15.3 ± 9.1	119 ± 15.8	101 ± 20.5		
RFD (mN × ms^{-1})	920 ± 100	209 ± 1.7	377 ± 35.6	309 ± 53.5		
sRFD (mN/mm2 × ms^{-1})	231 ± 16.1	138 ± 5.6	329 ± 36.5	276 ± 54.9		
RFD/PF (%)	11.7 ± 0.9	10.4 ± 0.8	2.77 ± 0.13	2.76 ± 0.20		
FI (%)	26.7 ± 5.2	9.9 ± 9.9	64.9 ± 3.79	75.9 ± 9.83		

EDL: extensor digitorum longus; SOL: soleus; CON: control condition; FAS: fasting condition;PF, peak force; P0: specific force; RFD; rate of force development; FI: fatigue index. Notes: ss, strain effect $p<0.01$; cc, condition effect $p<0.01$; ccc, condition effect $p<0.001$; mm, muscle type effect $p<0.01$; SC strain x condition interaction $p<0.05$; CM, condition x muscle type interaction $p<0.05$; SMT, strain × muscle type × time interaction $p<0.05$.

Biology Open • Supplementary information
